# Incorporating atezolizumab in the adjuvant setting of non-small cell lung cancer: key discussion points from an expert multidisciplinary panel by Italian Association of Thoracic Oncology

**DOI:** 10.3389/fonc.2022.971042

**Published:** 2022-07-22

**Authors:** Filippo de Marinis, Ilaria Attili, Cesare Gridelli, Fabiana Cecere, Carlo Curcio, Francesco Facciolo, Lorenzo Spaggiari

**Affiliations:** ^1^ European Institute of Oncology, IRCCS, Division of Thoracic Oncology, Milan, Italy; ^2^ ’S.G. Moscati’ Hospital, Division of Medical Oncology, Avellino, Italy; ^3^ Regina Elena National Cancer Institute, IRCCS, Oncology 1, Rome, Italy; ^4^ Department of Thoracic Surgery, Monaldi Hospital, Naples, Italy; ^5^ Thoracic Surgery Unit, Regina Elena National Cancer Institute, IRCCS, Rome, Italy; ^6^ European Institute of Oncology, IRCCS, Division of Thoracic Surgery, Milan, Italy; ^7^ Department of Oncology and Hemato-Oncology, Università degli Studi di Milano, Milan, Italy

**Keywords:** atezolizumab, immunotherapy, adjuvant, perioperative treatment, surgery

## Introduction

Despite recent advances in the advanced setting, lung cancer remains the primary cause of cancer death worldwide. Non-small cell lung cancer (NSCLC) represents approximately 85% of overall lung cancer cases (1). About 25% of patients with NSCLC are diagnosed with an early-stage disease and are candidate to receive surgical treatment with curative intent (2). Unfortunately, although radical resections are performed, only less than half of these patients are really cured, whereas disease recurrence is observed in 50-60% patients at 5 years (3–5).

Historically, the addition of platinum-doublet chemotherapy in the perioperative setting, either adjuvant or neoadjuvant, led to a 5% global increase in 5-year overall survival (OS) as compared to surgery alone (6). Based on these data, four cycles of cisplatin-based treatment have been considered the standard adjuvant approach in patients with resected NSCLC whose primary tumors were 4 cm or more in their greatest diameter (T≥ 4 cm) or had nodal involvement after adequate nodal dissection (stage IB-IIIA according to the 7^th^ American Joint Committee on Cancer -AJCC- TNM prognostic staging system) (7).

The adoption of the same treatment regimen in the neoadjuvant setting has historically been barely limited to patients with evidence of clinical or pathological nodal involvement, mostly N2, at mediastinal staging (stage IIIA N2, 7^th^ TNM edition) (8).

**Figure 1:**
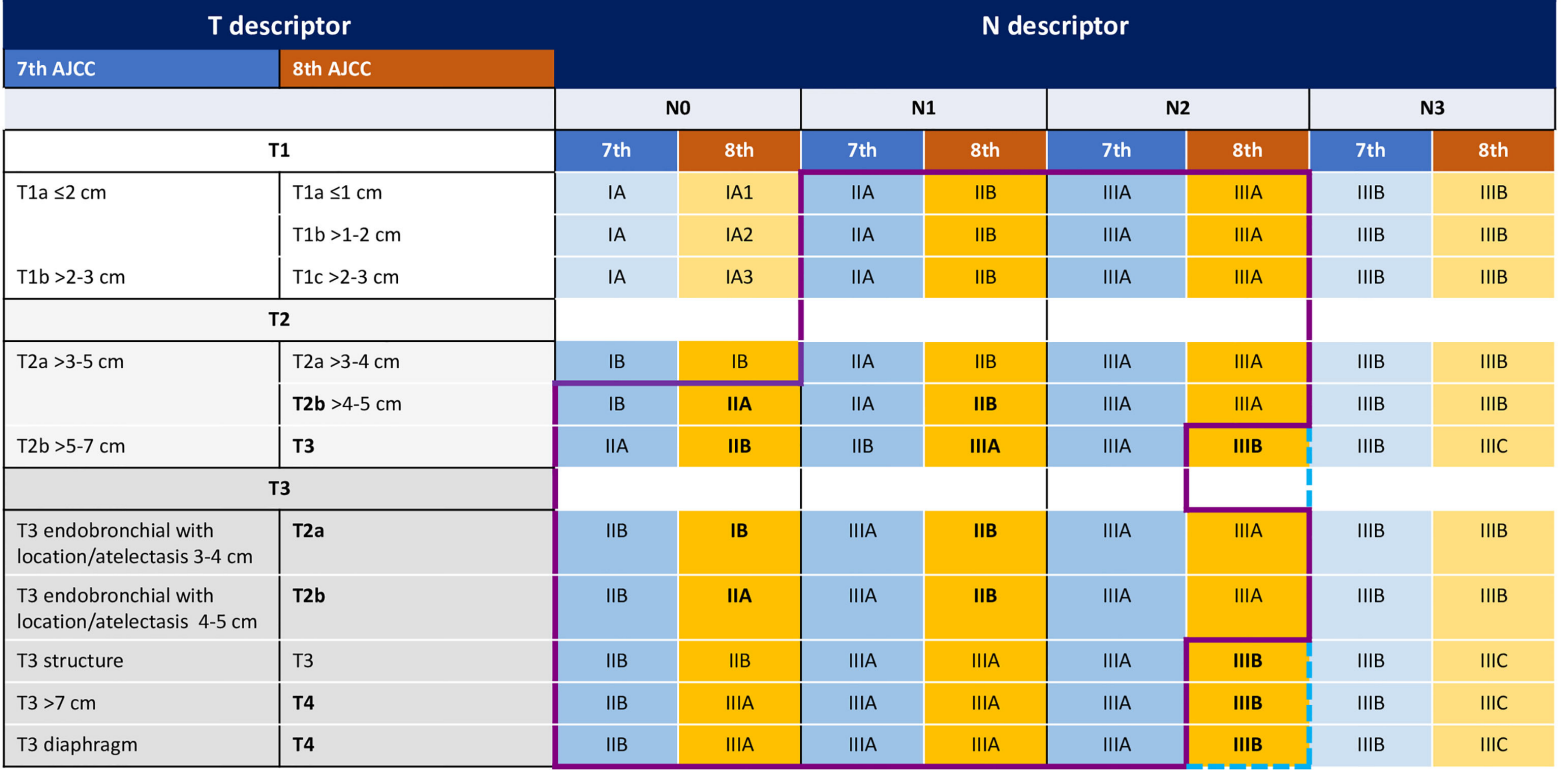
The figure shows the main changes in TNM categories from the 7^th^ to the 8^th^ AJCC TNM edition. Purple line indicates the patients included in the Impower010 trial according to the 7^th^ TNM, blue line indicates the patients included according to the 8^th^ TNM.

Despite these efforts to improve survival, more than 50% of patients recur within five years from the curative treatment. According to the novel 8^th^ AJCC TNM staging system, prognostic categories have been redefined, with 5-year OS rate ranging from 68% in stage IB to 36% in stage IIIA (3). Of note, the current staging system includes T3N2 tumors in stage IIIB category, which subgroup remains evaluable for curative-intent treatment (**Figure 1**).

With the aim to increase the cure rate of early-stage NSCLC, both molecular-based and immunotherapy based perioperative treatments are being evaluated in patients with resected tumors. Impressively, the administration of adjuvant osimertinib for 3 years in patients (stage IB-IIIA 7^th^ TNM) harboring common *EGFR* mutations reduced by 80% the probability of disease recurrence, regardless the use of adjuvant chemotherapy (9). On the same perspective, clinical trials are ongoing evaluating adjuvant targeted treatments in resected oncogene-driven tumors. In parallel, following the results obtained in the advanced disease, immune checkpoint inhibitors (ICIs) have been investigated in the perioperative setting. In this opinion article we aim to discuss the results obtained in the adjuvant setting of NSCLC with atezolizumab, in light of the recent regulatory approvals by Food and Drug Administration (FDA) and European Medicines Agency (EMA) and its application in clinical practice.

## Main evidence of adjuvant atezolizumab from the registrative randomized clinical trial

The IMpower010 was a multicenter phase 3 randomized clinical trial enrolling 1280 patients with completely resected stage IB (≥4 cm) to IIIA NSCLC (7^th^ TNM edition) between 2015 and 2018 (10). In this trial, patients received adjuvant atezolizumab 1200 mg every 21 days for 16 cycles (1 year) or best supportive care in a random assignment (1:1) after at least 1 cycle of adjuvant cisplatin-based chemotherapy. The primary endpoint was disease free survival (DFS), hierarchically tested as follows: DFS in stage II-IIIA PD-L1 positive (≥1%) population, DFS in all stage II-IIIA population, DFS in the intention-to-treat (ITT) population. At data presentation, with a median follow up of 32.2 months, 35/39/37% and 46/45/43% of DFS events occurred in the atezolizumab and BSC group in the three defined populations, respectively. DFS was significantly improved with atezolizumab compared to BSC in stage II-IIIA PD-L1 positive population (median NE vs 35.3 months, HR 0.66, 95% CI 0.50-0.88, p=0.004), and in all stage II-IIIA population (median 42.3 vs 35.3 months, HR 0.79, 95% CI 0.64-0.96, p=0.020). The third step of the hierarchical testing, DFS in ITT population, was not met, with HR 0.81 (95% CI 0.67-0.99, p=0.040) (10).

Based on these results, atezolizumab was the first ICI approved by FDA as adjuvant treatment for patients with completely resected stage II-IIIA NSCLC whose tumors had PD-L1 ≥1%.

Overall survival data were immature at data presentation, with HR 1.07 (95% CI 0.80-1.42) in the ITT population. In addition, according to the hierarchical testing, OS as secondary endpoint was not formally tested as DFS in ITT population did not meet statistical significance (10).

## Key discussion points for patient selection

Although treatment related adverse events with atezolizumab were mostly manageable (only 22% of grade 3 or 4 adverse events, 8% grade 3-4 immune-related adverse events) (10), the risk for immune-related and long-term toxicities of 1-year atezolizumab should be well balanced in the adjuvant setting, where a proportion of patients might be already cured. In this view, adequate patients’ selection is needed in order to avoid unnecessary treatment, as well as to increase the rate of cured patients or at least to prolong the time of relapse in high-risk patients.

### Multidisciplinary management

The first step for adequate selection of patients is a correct multidisciplinary management (11). Patients with early-stage lung cancer are mostly evaluated as first by the thoracic surgeon, whose role is crucial in different phases: the diagnosis, the staging, the cure. As per international guidelines, after NSCLC diagnosis, an adequate disease staging includes at least contrast-enhanced CT scan of chest and abdomen and a brain imaging (CT or magnetic resonance imaging MRI) (7). Patients who are candidate to surgical treatment should be also evaluated with 18FDG-positron emission tomography (PET) to exclude distant metastases and to investigate nodal status. Mediastinal nodal staging is also recommended with endobronchial ultrasound (EBUS) bronchoscopy and transbronchial needle aspiration (TBNA) to identify N positive tumors to exclude from surgery (confirmed pathological N3) or to propose for neoadjuvant treatment (e.g., confirmed pathological N2) (12). Following this complete evaluation, it is recommended that the treatment indication is endorsed by a multidisciplinary team composed of at least the thoracic surgeon, the medical oncologist, the radiation therapist, the pathologist and the pneumologist. In the absence of neoadjuvant treatment, patients undergoing complete resection should be evaluated in the same multidisciplinary context to select those who will benefit from adjuvant treatment. The nodal staging within surgical treatment remains to date one of the major issues to adequately select patients. Indeed, a very recent report from the ALCHEMIST study shows that among 2833 patients with resected stage IB (≥4cm)-IIIA (7^th^ TNM), only 53% had an adequate lymph node dissection (13). Patients in the IMpower010 trial were required to have mediastinal lymph node dissection (80%) or sampling (18%) at specified levels to be included in the study (10). Hence, over T dimensions, an adequate surgical treatment with appropriate nodal staging is required to identify patients with pathologically positive nodes who met criteria to receive adjuvant atezolizumab to potentially reproduce DFS results obtained within the clinical trial.

### Stage IB

Following the adoption of the 8^th^ TNM edition, the classification of stage IB tumors has changed and requires to be focused to warrant consistent considerations. Indeed, the main point is that those stages IB ≥ 4 cm (7^th^ TNM) included in the IMpower010 trial are actually classified as stage II tumors according to the 8^th^ TNM edition (14). Of note, reports on resected small NSCLC tumors with negative nodal status after adequate mediastinal nodal dissection, showed 5-year OS of 83-89% (15, 16), therefore the risk-benefit ratio of any adjuvant treatment with the objective to further increase survival would be very challenging in this setting. Hence, patients with resected stage IB NSCLC (without *EGFR* mutation) according to the current TNM edition are not candidate to receive any adjuvant treatment, unless future studies will investigate this particular setting.

### Clinical and biological features

Resected stage IIIA (40% of patients in the IMpower010 trial) who did not receive any neoadjuvant treatment (e.g., occult N2) are considered as very high-risk category for disease relapse. However, clinical features of patients should always be evaluated in the multidisciplinary context to decide for adjuvant treatment, including ICI. Indeed, patients in the IMpower010 trial were required to receive standard adjuvant cisplatin-based chemotherapy (10).

In clinical practice, a proportion of patients who are surgically resected for NSCLC present with major comorbidities or with impaired respiratory function (e.g., after pneumonectomy or in patients with COPD), or are elderly patients. Those patients would not be good candidates for standard chemotherapy doses, and in clinical practice might receive no indication for adjuvant treatments, or even receive carboplatin-based chemotherapy at lower doses. In these cases, the applicability of adjuvant atezolizumab remains limited. Furthermore, the potential immune-related adverse events of 1-year ICI, including pneumonitis, would be well balanced in patients who are potentially already cured and have impaired residual respiratory function.

In parallel, biological features should be included in patients’ evaluation. To date, few data are available about the efficacy of adjuvant ICI in patients with driver gene alterations. In the advanced disease, mono-immunotherapy showed no efficacy in the majority of driver-mutant NSCLC, especially those not related to smoking (17). Patients with EGFR or ALK positive tumors were included in the IMpower010 trial, with no benefit of atezolizumab compared to BSC in these subgroups. In the light of future options with targeted adjuvant treatments for those patients, atezolizumab use is limited in this setting. Conversely, further investigation on biological features (molecular alterations, co-mutations, tumor mutational burden, immune microenvironment) would be helpful to identify those patients, even with smaller tumors, at higher risk for recurrence, who might deserve the addition of adjuvant atezolizumab. In this view, a very recent report showed solid-predominant stage I adenocarcinoma as having higher disease recurrence rate compared to non-solid tumors (50% vs 20% at 4 years). Those tumors were also found to have higher immune cells infiltrate, higher PD-L1 expression and TMB, with those features associated to higher risk of recurrence (18). These findings suggest the potential benefit of adjuvant immunotherapy in this group.

In addition, the role of ctDNA was evaluated in the IMpower010 trial: the presence of post-surgical ctDNA (before chemotherapy) was associated with worse prognosis, and the use of atezolizumab had greater DFS benefit in this subgroup compared to observation (19.1 vs 7.9 months) (19).In this view, the evaluation of minimal residual disease (MRD) through NGS analysis might be helpful to define the presence of micro-metastatic disease and select patients for adjuvant treatments.

### PD-L1

The secondary endpoints of the IMpower010 study included DFS in patients with stage II-IIIA tumors expressing PD-L1 on 50% or more (≥50%) tumor cells. This subgroup included 229 patients overall, who had greater magnitude of DFS benefit with atezolizumab compared to BSC (median NE vs 35.7 months, HR: 0.43, 95% CI 0.27-0.68) (10).

Patients with stage II-IIIA whose tumors had PD-L1 expression between 1% and 49% (PD-L1 1-49%) were 247. In this subgroup, as well as in PD-L1 negative subgroup, investigated in a *post-hoc* exploratory analyses, no clear advantage with adjuvant atezolizumab over BSC was seen (HR 0.87, 95% 0.60-1.26; HR 0.97, 95% CI 0.72-1.31, respectively) (10).

Based on these results, in April 2022, the EMA adopted the indication for atezolizumab monotherapy as adjuvant treatment after complete resection and adjuvant platinum-based chemotherapy, for patients with high-recurrence risk NSCLC with PD-L1≥50% and absence of EGFR or ALK driver gene alterations.

## Discussion

In the last decades, no advances were obtained in the adjuvant setting of NSCLC, with about half patients relapsing after curative surgery. In this view, medical oncologists applied adjuvant chemotherapy whenever possible in high-risk patients, often with underdosing regimens in unfit patients, with the aim to reach at least the 5% OS increase demonstrated in previous metanalyses (6). In this view, the potential decrease in disease recurrence rate by 34% demonstrated with the addition of 1-year adjuvant atezolizumab in PD-L1 positive stage II-IIIA represents a remarkable step forward. As often debated, DFS represents a surrogate endpoint for OS in the adjuvant setting and this has always represented a reason to consider with caution DFS positive results while waiting for final OS data (20). To corroborate this doubts, early results from the KEYNOTE-091/PEARLS trial were presented. In this phase III randomized study, 1-year pembrolizumab showed significant DFS improve in all-comers populations of resected stage IB (≥4 cm)-IIIA NSCLC (7^th^ TNM edition) (median 53.6 vs 42.0 months; HR 0.76, 95% CI 0.63-0.91, p = 0.0014) but not in PD-L1 high subgroup (however with median not reached in either arm) (21).

Despite the limitations of subgroup analyses, the EMA indication in PD-L1 high tumors represents in our view, a valid option to select patients for adjuvant atezolizumab in the absence of more solid data on long-term DFS and potential OS impact.

To date, it is unknown whether the 1-year duration of adjuvant treatment is enough, too short, or too long. Longer follow-up, together with considerations on long-term adverse events and financial costs, will help to define this aspect. Furthermore, no data on the efficacy of ICI rechallenge at disease recurrence are available in patients who receive atezolizumab in the adjuvant setting. In this context, the timing and the pattern of relapse (22), as well as PD-L1 levels and potentially a rebiopsy to assess tumor biology at recurrence, will help to define ICI-resistant or sensitive tumors.

Another point to raise is the role of adjuvant post-operative radiotherapy (PORT) in pN2 NSCLC, that has been recently questioned by the negative results of the Lung-ART and PORT-C studies (23, 24). However, it is still uncertain whether there might be any patients who can benefit from PORT. As most adjuvant trials with immune checkpoint inhibitors did not allow the use of PORT, this remains a field of potential investigation (25).

## Author contributions

Conceptualization: FdM and LS. Methodology: all authors. Data collection: all authors. Writing-original draft preparation: IA and FdM. Writing-review and editing: all authors. Visualization: IA and FdM. Supervision: FdM and LS. All authors have read and agreed to the published version of this manuscript.

## Funding

This work was partially supported by Associazione Italiana di Oncologia Toracica (AIOT).

## Acknowledgments

This work was partially supported by the Italian Ministry of Health with “Ricerca Corrente”, “5x1000”.

## Conflict of interest

FdM has served in a consultant/advisory role for Astra Zeneca, Boehringer Ingelheim, Bristol-Myers Squibb, Celgene, Merck Sharp & Dohme, Novartis, Roche Genentech, Takeda and Pfizer, outside the submitted work. CG received honoraria as speaker bureau or advisory board member or as consultant from MSD, BMS, Roche, AstraZeneca, Novartis, Pfizer, Menarini, Boehringer, Karyopharm and Eli Lilly. The authors have no other relevant affiliations or financial involvement with any organization or entity with a financial interest in or financial conflict with the subject matter or materials discussed in the manuscript apart from those disclosed.

The remaining authors declare that the research was conducted in the absence of any commercial or financial relationships that could be constructed as a potential conflict of interest.

## Publisher’s note

All claims expressed in this article are solely those of the authors and do not necessarily represent those of their affiliated organizations, or those of the publisher, the editors and the reviewers. Any product that may be evaluated in this article, or claim that may be made by its manufacturer, is not guaranteed or endorsed by the publisher.
